# ADRD Caregivers’ Hesitancy to Receive Preparedness Program: Caregiving Context and Resilience

**DOI:** 10.1093/geroni/igaf122.3524

**Published:** 2025-12-31

**Authors:** Emily Killian, Sato Ashida, Nicholas Ostrem, Aaron Seaman

**Affiliations:** University of Iowa, Iowa City, Iowa, United States; University of Iowa, Iowa City, Iowa, United States; University of Iowa, Iowa City, Iowa, United States; University of Iowa, University of Iowa, Iowa, United States

## Abstract

Preparedness is paramount for caregivers of persons living with dementia (PLWD) who face unique challenges in disaster situations, yet some are hesitant to participate in programs that help them develop emergency plans. Understanding factors associated with hesitancy can guide recruitment and program adaptation. Caregivers of PLWD randomized into the intervention arm (n = 112) of the Disaster PrepWise-Caregiving trial completed surveys and received a preparedness program. We compared caregivers who expressed hesitancy (n = 16) with those who did not (n = 96) on caregiving context, burden, self-efficacy, and resilience using t-tests and descriptive statistics, and analyzed post-study interviews guided by the PRISM framework and interventionists’ notes. Hesitant caregivers reported lower caregiving demands, providing fewer weekly hours (31% < or equal to 8 hrs/week vs. 16%), shorter caregiving duration (38% < 2 years vs. 19%), and were more likely to care for PLWD in mild FAST stages (25% vs. 8%). Hesitant caregivers reported significantly lower Zarit burden scores (M = 2.19) than non-hesitant caregivers (M = 2.95, p = 0.015), but higher self-efficacy to manage dementia symptoms (p = 0.037) and higher CD-RISC perseverance (p = 0.032) and adaptability (p = 0.018) scores. Qualitative data reinforced these patterns: hesitant caregivers describe preparedness as currently unnecessary because care demands were not severe, or they felt equipped to cope through existing supports. Findings highlight that caregivers of individuals in earlier stages may not perceive preparedness as a current priority, indicating need for primary prevention messaging and recruitment strategies that emphasize planning benefits within ADRD caregiving context. Future research should identify strategies that motivate engagement across the spectrum of perceived need.

